# The symbiotic experiences of residents with and without dementia co-living in Taiwan’s long-term care facilities: a phenomenological study

**DOI:** 10.1186/s12877-024-05205-9

**Published:** 2024-07-17

**Authors:** Chan-Chuan Fang, Yi-Hsun Liu, Shu-He Huang

**Affiliations:** 1 Department of Nursing, College of Nursing, National Yang Ming Chiao Tung University, No. 115, Sec. 2, Linong St. Beitou Dist. 112, Taipei, Taiwan, R.O.C.; 2 Nursing Home, Changhua Hospital, Ministry of Health and Welfare, No. 80, Sec. 2, Zhongzheng Rd., Puxin Township, 513 Changhua County, Taiwan, R.O.C.

**Keywords:** Residents, Dementia, Long-term care facility, Symbiosis

## Abstract

**Background:**

In Taiwan, residents with and without dementia mostly co-live in long-term care facilities. The behavioral and psychiatric symptoms of dementia residents often pose challenges for others living together. This study explored the symbiotic experiences of residents without dementia co-living with those with dementia in long-term care facilities in Taiwan to present their experiences of living together.

**Methods:**

This was a cross-sectional descriptive study with a phenomenological design. Semi-structured face-to-face interviews were conducted with 30 residents without dementia from three long-term care institutions in Taiwan. Colaizzi’s data processing steps were used for analysis.

**Results:**

The analysis of interview transcripts revealed that the experiences of residents who lived with those with dementia were that of a “symbiosis.” Three core themes were found: *“the impact of co-living*,*” “facing difficulties and coping*,*”* and *“companionship and reciprocity.”* This study showed that residents without dementia may be affected by the behavioral and psychiatric symptoms of residents with dementia when co-living in long-term care facilities. However, there are also positive and mutually beneficial interactions between them. By helping people with dementia in their daily lives, residents without dementia feel happy and accomplished and their self-worth is enhanced. Furthermore, residents with dementia have more opportunities for social engagement and co-living interactions.

**Conclusion:**

These results can guide long-term care facilities without special care dementia units to support residents without dementia, reduce the interference of the behavioral and psychiatric symptoms of residents with dementia, and promote mutual benefits. However, these findings warrant further investigation.

## Background

The number of people with dementia worldwide was estimated to exceed 55 million by 2023, with nearly 10 million new cases each year [1]. In Taiwan, 7.54% of the population over the age of 65 years and 20% over the age of 80 years have dementia. Based on the 5-year prevalence rate, the population with dementia in Taiwan is estimated to increase to 460,000 by 2031, with two out of every 100 people having dementia [2].

Dementia is a progressive degenerative disease; it develops from a mild intellectual disability, gradually progressing to a decline in the abilities of daily life activities in the middle stage, to complete dependence on others in the late stage. Throughout their illness, 97% of people with dementia are affected by one or more behavioral and psychological symptoms of dementia (BPSD) [3], including psychosis, delusions and hallucinations, agitation (e.g., irritation, arguing and complaining, pacing, inappropriate screaming, crying, repetitive questioning, refusing care, running away from home), aggression (behavior or speech), depression, anxiety (e.g., worrying or shadowing), apathy, disinhibition (social and sexual misconduct), motor disturbance (e.g., wandering, rummaging), and night-time behaviors (e.g., waking up in the middle of the night), as well as abnormal appetite and eating problems. The type of BPSD is associated with the stage of dementia [4]. When people with dementia develop BPSD, training formal or informal caregivers to provide person-centered care or improve communication skills to deal with BPSD and using dementia care mapping (DCM) as an assessment tool are the most effective ways to reduce the care burden [5].

Currently, dementia care is available at home, in the community, and in institutions. Patients in the early stages of dementia can be cared for at home or in the community. In later stages, when patients must rely on others, it causes pressure and burdens on caregivers, and the assistance of professional caregivers in long-term care institutions is required. Due to the complexity of behavioral care issues with dementia, the concept of dementia specialty care units (DSCUs) has become a strategy for the management of dementia care [6]. DSCUs are designed to reduce inappropriate antipsychotic use, pressure injuries, feeding tubes, physical restraints, and hospitalization [7]. However, the ownership status, geographic region, and nurse staffing of the facility affect the setup of DSCUs, and only about 15% of nursing homes in the United States have DSCUs [8].

Similarly, DSCUs in Taiwan are still promoted but are mostly limited to government operations. Dementia care institutions in Taiwan include integrated dementia centers, community-based dementia service stations, daycare services, group homes, and residential long-term care institutions (including welfare institutions for older adults, institutions for the disabled, nursing homes, veteran homes, and ministerial hospitals). There are only 2,437 beds in dementia special care units in Taiwan [9] which is far from meeting the demand of people with dementia. Therefore, when people with dementia require long-term care in institutions, most have to depend on general long-term care institutions. According to a recent survey, the prevalence of all-cause dementia in Taiwan’s long-term care facilities is 87.1% [10]. Additionally, admission to long-term care facilities, except for a few social welfare placements, is paid by the residents themselves. Due to financial considerations, most residents cannot afford the costs of single rooms. The business strategy of private long-term care facilities tends to focus on shared rooms with fewer single rooms.

Thus, dementia residents are required to co-live with other residents without dementia. Even those with private rooms still encounter other residents with dementia in public activity spaces and can be challenged by their BPSD. Previous studies have mostly examined the experiences of residents with dementia living in institutions [11], their families, and caregivers [12], as well as their quality of life in institutions. However, few have focused on the lived experiences of residents without dementia who co-live with patients with dementia in long-term care institutions. Cheng described the positive and negative cohabitating experiences of cognitively intact residents and residents with dementia in long-term care facilities [13]. Taiwan has evolved from an aging society to an aged society and will enter a super-aged society in 2025. This, together with low fertility rates, has led to a reduction in care staff and the increasing cost of long-term care has also placed pressure on government finances [14]. In response to the changes in social structure, the concept of co-housing communities has been gradually applied to care and means “caring for the community” with symbiotic and shared values. Japan entered the super-aged society earlier than Taiwan and began building a “Community Integrated Care System” in 2012. The care space is a symbiotic care structure that no longer distinguishes between senior citizens and the disabled and includes the general population. Long-term care facilities comprise micro-societies. Exploring the life experiences of people with dementia (disability) and without dementia (function is normal compared to dementia patients) will help us understand how needs have evolved over time and can be used as support or suggestions for relevant policy improvements.

Therefore, this study aimed to understand the experiences of residents without dementia living with those with dementia in long-term care institutions.

## Methods

### Study design and setting

This was a cross-sectional descriptive study using a qualitative phenomenological analytical approach. Husserl’s phenomenology emphasizes the method of returning to the facts, restoring the phenomena, constantly questioning, and understanding the “essence” of experiential events through intuitive insight, using “vernunftiger ausweisung” and “bracketing” to describe overall life experiences [15].

This method was used to study the essential meaning of the lived experiences of a particular phenomenon by interviewing residents without dementia and understanding the possible impact of living with residents with dementia in long-term care facilities.

### Participants and recruitment

Participants were recruited through purposive sampling from three mixed-care long-term care facilities in central Taiwan between May 28, 2017, and November 15, 2017. These facilities are privately operated multi-story buildings. The total number of beds is between 100 and 219 per facility, with occupancy rates greater than 90%. Men and women do not live in the same rooms, and room types include single, double, triple, and sextuple rooms. The staff, including nurses, are responsible for medical care and related administrative duties. Nurse aids assist residents with disabilities in their physical cleansing and daily activities such as bathing, changing positions, and tube feeding/oral feeding. Functioning residents take care of themselves personally. The ratio of nurse/resident is 1:15, and the ratio of nurse aid to resident is 1:5 ~ 8.

First, the researchers identified potential research participants with the help of nursing staff from the long-term care facilities from residents without dementia who co-lived for at least one month with those with dementia and BPSD. Thus, residents without dementia were solicited to participate in the study. To exclude those with cognitive impairments who had not been diagnosed with dementia, it was necessary to assess their Mini-Mental State Examination (MMSE) scores and confirm with staff that the participants did not have any unstable mental illnesses. This ensured that there were no disruptions in the interview process and in the analysis of the results. The participants were required to communicate clearly, regardless of age.

The inclusion criteria were as follows: (1) close contact with residents with dementia or living in the same room or event space; (2) MMSE score met the criteria (MMSE ≧ 24 points = more than six years of education; MMSE ≧ 20 points = less than six years of education; MMSE ≧ 17 points = no education); and (3) fluent communication in Chinese and Taiwanese, without expression barriers. The exclusion criteria were as follows: (1) mild to severe depression, mental illness, and unstable condition after treatment and (2) hearing difficulty without hearing aids or inability to communicate with hearing aids. Participants who satisfied all inclusion criteria were informed about the study, including its purpose and method, and given an assurance of data confidentiality. They had the right to choose to participate or drop out. The number of cases was recorded until data reached saturation. The final sample comprised 30 residents without dementia. Studies show that behavioral and psychological symptoms are prevalent in the cognitively impaired older population [16]. Therefore, after the interviews, the researchers confirmed with staff that the participants’ answers were oriented specifically to residents with dementia.

### Data collection

Semi-structured interviews were conducted. To truly present the experience of residents without dementia co-living with residents with dementia and to avoid the stigma or confrontation that may affect the study’s results, we took the following measures: Not only did we confirm that the participants met the criteria before the interview, but we also confirmed the interview content with staff after the interview to ensure that the interview guidelines (Table 1) did not bias the results by focusing on getting along with residents with dementia.

The interview guidelines were formulated from the researcher’s experience and the opinions of five experts, including nursing scholars, clinical psychosomatic physicians, neurologists, and clinical psychologists. Primary interviews were conducted with individual participants in quiet, undisturbed meeting rooms. Each interview lasted between 45 and 60 min and was tape-recorded.

**Table 1:** Interview guide for residents who are cognitively intact

**1**	How long have you been living here?
**2**	How do you get along with the other residents living here?
**3**	What impresses you when you spend time with other residents?
**4**	What makes you happy living here with other residents?
**5**	What is it about living here that makes you unhappy with other residents?
**6**	Has there been a situation where you were affected or disturbed by other residents, and could you describe the situation at that time?
**7**	Have you ever had a conflict with other residents, or have you seen a conflict between other residents? If so, describe the situation.

### Data analysis

The study data were systematically analyzed following Colaizzi’s 7-step analysis method: Interview tapes were listened to carefully and transcribed verbatim, meaningful sentences were extracted from each transcript, and meanings from sentences were extracted and coded. The codes with the same attributes were clustered into themes, the results were integrated and described in depth, and the essential structure of the phenomenon was formed. To ensure that the results reflected the participants’ experiences, we sent each transcript and result to the participants to affirm the findings [17].

### Trustworthiness

The credibility, transferability, dependability, and confirmability of this study were used to ensure trustworthiness [18]. Individual interviews were conducted by YHL, who worked in a long-term care institution and has practical interview experience. The other researchers also had clinical experience in providing care to older adults with and without dementia and had studied qualitative research methods and interview techniques. Before the interview, the researchers screened for potential biases such as positions, assumptions, and preconceptions and constantly reminded the participants not to let subjective consciousness influence their thoughts. The raw data, processes, and analytical notes, including audio recordings, interview transcripts, and coding schemes, were appropriately stored in computers to maintain audit trails and provide a basis for understanding the relevance and significance of the data. Furthermore, through a detailed description and conceptualization of the background conditions of the phenomena, the transferability of the data was enhanced, which can be used as a reference for other researchers conducting similar research, ensuring that the research results are widely used as a reference for clinical practice. The interpretation of the data was verified by three participants who provided further clarification and confirmed the accuracy of the transcription and observations. This increased the trustworthiness and credibility of the research data.

### Ethics

This study was approved by the Human Research and Ethics Committee of Yang-Ming University (Institutional Review Board [IRB] approval number: 106,006 F). The research process adhered to the principles of altruism, non-harm, and confidentiality. The researchers recruited participants, informed them about their rights, and initiated interviews after they agreed to participate and signed the consent form. Additionally, original files, such as relevant audio recordings and documents, were only used by the researchers when analyzing data, and the research results were presented anonymously.

## Results

### Participant characteristics

Thirty participants residing in long-term care facilities were included. There were 25 (83.3%) women and five (16.7%) men, with a mean age of 82 years (range = 71–94). Most participants were widows (*n* = 25, 83.3%), followed folk beliefs (*n* = 12, 40.0%), and had an educational level of primary school (*n* = 17, 56.7%). The mean years of living in long-term care facilities were 4.29 years (range = 0.25–16.8 years), most stayed from 1 to 3 years (*n* = 14; 46.7%) and lived in 6-person rooms (*n* = 10; 33.3%). The mean MMSE score was 25 points, and 21 participants had high MMSE scores (range = 24–27). The mean score for activities of daily living (ADL) was 75; 17 participants had ADL scores between 81 and 100; the mean score for instrumental activities of daily living (IADL) was 12, and 22 participants had an IADL score of less than 15 (see Table 2).

**Table 2:** Characteristics of residents without dementia

Number	Sex	Age	Educational level	Marriage	Religion	Total length of stay (Months)	MMSE	ADL	IADL	Number of beds
1	Male	85	Elementary school	Widowed	I-Kuan Tao	15	28	95	10	3
2	Female	76	Elementary school	Widowed	Buddhism	78	26	90	9	2
3	Female	85	Elementary school	Widowed	Buddhism	168	28	100	21	1
4	Female	87	Junior high school	Widowed	Buddhism	207	26	50	8	3
5	Female	90	Elementary school	Married	Buddhism	61	25	95	21	2
6	Male	85	Illiteracy	Widowed	Buddhism	202	24	95	12	3
7	Female	71	Junior high school	Widowed	Folk beliefs	35	25	100	13	6
8	Female	93	Illiteracy	Widowed	Buddhism	35	24	80	12	6
9	Female	85	Elementary school	Widowed	I-Kuan Tao	21	27	90	12	3
10	Female	85	Elementary school	Widowed	Folk beliefs	7	26	25	5	6
11	Female	94	Elementary school	Widowed	Buddhism	121	27	100	18	1
12	Female	85	Elementary school	Widowed	Folk beliefs	6	20	25	5	6
13	Female	74	Elementary school	Widowed	I-Kuan Tao	7	24	85	9	2
14	Female	77	Elementary school	Divorce	Folk beliefs	9	25	75	8	6
15	Male	80	Illiteracy	Married	Folk beliefs	4	20	20	7	6
16	Female	85	Illiteracy	Widowed	Taoism	54	25	90	12	3
17	Female	81	Elementary school	Widowed	Christian	17	27	75	7	6
18	Female	83	Elementary school	Widowed	Buddhism	3	26	95	17	2
19	Female	87	Elementary school	Widowed	Buddhism	21	24	35	4	6
20	Female	87	Illiteracy	Widowed	Irreligious	29	25	90	10	6
21	Male	84	Elementary school	Widowed	Folk beliefs	30	24	70	8	6
22	Female	72	Elementary school	Widowed	Folk beliefs	21	24	100	17	2
23	Female	88	Elementary school	Widowed	Folk beliefs	21	28	70	7	2
24	Female	90	Elementary school	Widowed	Folk beliefs	19	24	50	10	2
25	Female	78	Bachelor	Divorce	Buddhism	202	28	100	23	1
26	Man	77	high school	Unmarried	Taoism	3	28	100	23	2
27	Female	71	Bachelor	Widowed	Folk beliefs	72	24	95	15	2
28	Female	78	Illiteracy	Widowed	Folk beliefs	57	20	100	18	3
29	Female	85	Illiteracy	Widowed	Folk beliefs	3	20	25	18	3
30	Female	74	Junior high school	Widowed	I-Kuan Tao	19	25	35	5	3

**Notes**: MMSE, Mini-Mental State Examination; ADL, activities of daily living; IADL, instrumental activities of daily living

### Core themes

**Fig. 1:**
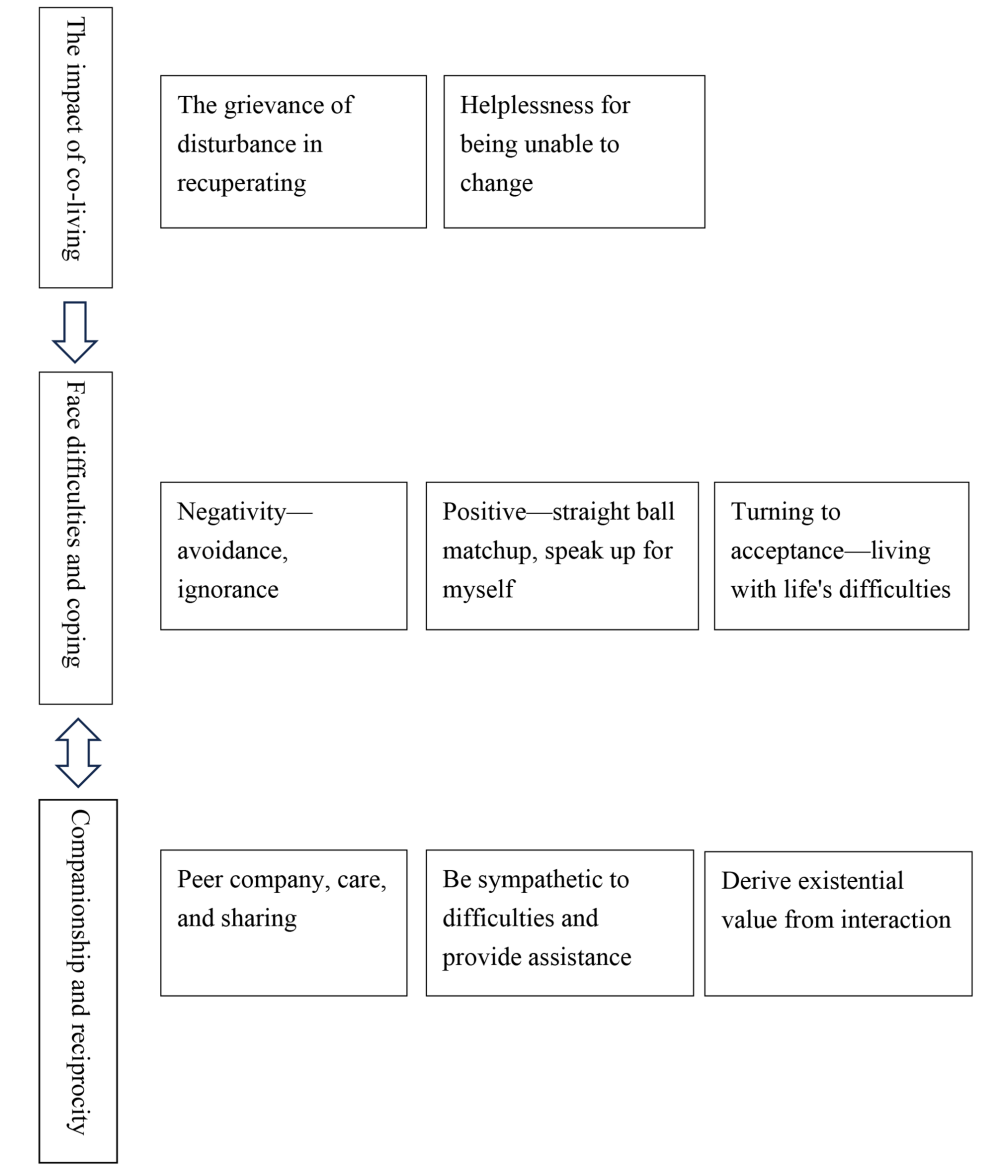
Residents with and without dementia co-living in long-term care facilities

Three main topics emerged from the analysis (1) *“The impact of co-living*,*”* (2) *“Face difficulties and coping*,*”* and (3) *“Companionship and reciprocity.”* Each theme represents the experience of residents without dementia co-living with dementia, as shown in Fig. 1. Table 3 provides an example of the process of data analysis and processing.

**Table 3:** Example of final themes, sub-themes, and codes

Interview transcript	Code	Sub-theme	Theme
*“Several times*,* I saw her enter my room. Then my shoes went missing and that scared me. I told her family*,* ‘Your mom is here to be taken care of*,* just like me*,* so how would you feel if you were in my shoes and felt insecure?’”*	Feeling unsafe about the odd behavior of the residents with dementia	The grievance of disturbance in recuperating	The impact of co-living
*“Sometimes*,* I feel uncomfortable. But where could I move to? I can’t afford to live in one bedroom. There wasn’t an affordable room available so I couldn’t part with her.”*	Unable to change the situation of co-living with dementia	Helplessness for being unable to change	
*“He has a foul mouth*,* criticizes*,* and always scolds others. Although I have heard the scolding*,* I choose to ignore it because I am in my 80s and my time is limited. …so his actions don’t affect my life.”*	Ignore the abnormal behavior of residents with dementia	Negativity—avoidance, ignorance	Face difficulties and coping
*“She’s noisy… Ah*,* I’m afraid of being noisy. … It’s noisy day and night*,* and I am disturbed when I take a nap*,* I am a light sleeper. … It’s really noisy. … I go to inform the leader. … Ask the attendant to move her out of the room.”*	Intolerance of dementia-related BPSD behavior, requiring staff to deal with it	Positive—straight ball matchup, speak up for myself	
*“Everyone is in the same boat. … Everyone lives together because of fate. His daughter asked me if he had disturbed me. I said it didn’t matter; we owed each other.”*	Everything is fate	Turning to acceptance—living with life’s difficulties	
*“If she drinks too much water*,* she may choke. …I quickly went to sit behind her and said*,* ‘Be careful! You have to take a break before drinking so you don’t choke…’”*	Caring for the residents with dementia	Peer company, care, and sharing	Companionship and reciprocity
*“I reflected on myself and told myself to forgive her. She must have some problems…that’s why she was like this… I will perhaps be like this in the future. …Put yourself in other people’s shoes.”*	Believe that the residents with dementia behave unintentionally.	Be sympathetic to difficulties and provide assistance	
*“If no one feeds her*,* I will provide for her; I treat them as if they were our own parents. …Some of them eat messily*,* but I feed them*,* and they eat the meal cleanly. …she may not prefer it when others serve her. …I feed her*,* and she is pleased …after I feed (him)*,* I feel relieved*,* and then I return for my meal. I do it every day.”*	Helping other residents with dementia feel a sense of accomplishment	Derive existential value from interaction	

#### The impact of co-living

In residential long-term care facilities without dementia special care units, people with dementia who request accommodation may share a room with residents without dementia depending on bed availability. Even if they do not live in the same room, they are likely to inhabit the same public spaces as residents without dementia. Therefore, residents without dementia may be disturbed by the varying degrees of BPSD of those having dementia, which may lead to difficulties in getting along in daily living in long-term care institutions as well as feelings of helplessness because of the unchangeable situation.

### The grievance of disturbance in recuperating

Residents with dementia and BPSD can experience negative impacts on their daily lives owing to the symptoms that include delusions, repetitive behaviors, sleep problems, restlessness, arguing, complaining, loitering, and aggressive behaviors. Such symptoms can disturb the everyday life and sleep of residents in an institution. For instance, the continuous noise made by residents with dementia can affect the quality of sleep and calmness of the environment, agitation can disrupt group harmony, and delusions can lead to misunderstandings with other residents.

Residents without dementia described how they were affected by the behavior of those with dementia:

**Case 17:**

*“I feel so much suffering*,* not just from the two people present*,* but also from other people who keep crossing their legs*,* patting their heads*,* slapping the bed rail*,* and not sleeping. …She wanted to call the nurse aide and shouted*,* ‘Ah Mei*,* Ah!’…She couldn’t find her*,* so she walked back and scratched the soles of my feet. …Her mouth kept whirring and whirring*,* giving me a headache every two hours; my brain hurt because of it.”*

**Case 26:**

*“Several times*,* I saw her enter my room. Then my shoes went missing and that scared me. I told her family*,* ‘Your mom is here to be taken care of*,* just like me*,* so how would you feel if you were in my shoes and felt insecure?’”*.

### Helplessness for being unable to change

Residents with dementia often exhibit uncontrollable and disruptive behaviors that cause grievances among those without dementia who attempt to communicate with them. However, because of their limited ability to understand their BPSD, communication is often ineffective. Consequently, those without dementia have no choice but to give up. Unfortunately, they may have to continue living with residents with dementia if there are no other suitable rooms in the institution or if they cannot afford a single room. This situation can be distressing, and residents described the helplessness they feel when forced to live with people with dementia:

**Case 30:**

*“I tried to explain*,* but how would I make her understand? She didn’t listen. …It was like talking to a wall.”*

**Case 5:**

*“Sometimes*,* I feel uncomfortable. But where could I move to? I can’t afford to live in one bedroom. There wasn’t an affordable room available so I couldn’t part with her.”*

#### Face difficulties and coping

When living together, residents without dementia may face dilemmas interacting with their co-residents with dementia. Some may choose to avoid them, ignore their BPSD, or seek help and self-protection against infectious diseases while safeguarding their belongings. Others may actively protest, express dissatisfaction, refuse to tolerate, bravely resist, or ask them to move out of the room and live separately. However, some residents choose to change their perspectives, adapt to the situation, and accept living with those with dementia to repay karma or as a predetermined fate. It is essential to understand that the troubles caused by those with dementia are not a reflection of the person and to calmly face and adapt to living with them.

### Negativity—avoidance, ignorance

To prevent BPSD from affecting their lives, residents without dementia may choose to ignore or avoid noisy, repetitive, or wandering behaviors displayed by those with dementia. This helps maintain a peaceful environment and prevent potential conflicts.

The residents explained how they deal with the disturbances caused by other residents with dementia without actively engaging with them.

**Case 3:**

*“It’s just that she forgot where she put her things. …She always had a suspicion that someone was stealing from her*,* and now this behavior is getting worse. …Nobody dared to go into her room unless they were called for some specific reason. …I didn’t want to go into her room.”*

**Case 4:**

*“He has a foul mouth*,* criticizes*,* and always scolds others. Although I have heard the scolding*,* I choose to ignore it because I am in my 80s and my time is limited. …so his actions don’t affect my life.”*

### Positive—straight ball matchup, speak up for myself

When residents without dementia were unable to tolerate the disruptive behavior of those with dementia, they may either confront them in person or seek staff assistance. Some residents who could not manage sharing a room may ask the person with dementia to move out or opt to move to a different room to reduce disturbances. Residents also described how they actively coped with distractions from residents with dementia by adopting a receptive approach to accepting and living with them.

**Case 15:**

*“He grabbed the remote control from me*,* but I refused to give it to him. In his anger*,* he started to insult us*,* even swear. …I asked him why he needed the remote when he had a TV in his room*,* but he continued to curse and demand that I bring it to him. …I ran away from him as he chased me*,* but I wasn’t afraid.”*

**Case 20:**

*“She’s noisy… Ah*,* I’m afraid of being noisy. … It’s noisy day and night*,* and I am disturbed when I take a nap*,* I am a light sleeper. … It’s really noisy. … I went to inform the leader. … Ask the attendant to move her out of the room.”*

### Turning to acceptance—living with life’s difficulties

Some residents without dementia found the BPSD of those with dementia disturbing. Some believed that they may have a karmic debt with residents with dementia and that living together and being interfered with was a necessary process of repaying it. Therefore, they chose to let their karma become good and faced the interference of living together. Others believe that it is their destiny to spend their old age together in institutions and that, given their advanced age, they could choose to accept their fate and ignore any disruptive behavior from residents with dementia.

**Case 13:**

*“It’s okay*,* anyway*,* it’s fate that we are together*,* whatever she wants (laughs)… As long as she’s happy (laughing)…. … Anyway*,* there is a fate together*,* it’s all fate. … I don’t want to move out.”*

**Case 30:**

*“Everyone is in the same boat. … Everyone lives together because of fate. His daughter asked me if he had disturbed me. I said it didn’t matter; we owed each other.”*

#### Companionship and reciprocity

When residents with and without dementia live together in institutions, they gradually recognize that they are essential companions in living, caring, and sharing. Those without dementia understand that the behavioral and mental conditions of residents with dementia are not intentional and may occur to them in the future. Participants wanted to assist them throughout their lives and found joy in helping others and forming mutual care relationships.

### Peer company, care, and sharing

Over time, when residents without dementia live with those with dementia, they begin to perceive residents with dementia as indispensable members of their lives. Residents living in these institutions become family members and provide emotional support to each other. They enjoy spending time together, caring for one another, sharing meals, and engaging in other daily activities.

**Case 18:**

*“She likes to eat (steamed buns). …It’s pitiful to see her screaming like this*,* but she doesn’t know how to get the steamed buns. …She has some (steamed buns)*,* but she doesn’t have enough. …Most of (the steamed bun) is mine*,* my portion. … I don’t have to eat so much! … I take them to her to eat.”*

**Case 14:**

*“She is unable to speak… If she feels familiar with you*,* she will sometimes give a high-five to you. She laughs*,* and I play with her. Just pat her like that… Nice to say hello to her. … I used to say to them*,* ‘Hey*,* my darlings.’ … Living together*,* it’s like people in the same family should take care of each other. “*.

**Case 17:**

*“If she drinks too much water*,* she may choke. …I quickly went to sit behind her and said*,* ‘Be careful! You have to take a break before drinking so you don’t choke…’”*.

### Be sympathetic to difficulties and provide assistance

Participants expressed understanding and empathy regarding the fact that residents with dementia may experience BPSD due to medical issues that can cause difficulties and distress. Participants adopt a caring and friendly approach towards their fellow residents with dementia and provide assistance when needed.

**Case 26:**

*“I reflected on myself and told myself to forgive her. She must have some problems…that’s why she was like this… I will perhaps be like this in the future. …Put yourself in other people’s shoes.”*

**Case 4:**

*“I have trouble falling asleep at night and during the day. Even the slightest sound wakes me up. My roommate opened and closed the drawer so loudly that I couldn’t help but wake up. I didn’t want to be disturbed by her. However*,* she has diabetes and craves cookies.”*

**Case 28:**

*“I poured tea and covered them with quilts. …When we lived together*,* we had to help each other. …If they can’t do it*,* I’ll do it for them. …If she can’t do the dishes*,* I’ll do it for her. …If she doesn’t have toilet paper to use*,* I’ll give it to her.”*

### Derive existential value from interaction

Participants believe that one stays in long-term care facilities for various reasons. Through helping and caring for residents with dementia in daily life, they perceive the needs and values of living, and through interaction and mutual companionship with older adults with dementia, they gain a sense of belonging and no longer feel lonely. This indicates a reciprocal relationship between the two parties.

**Case 7:**

*“Before coming here*,* I was not in good health. However*,* within a few months of moving here*,* I fully recovered. …I don’t want to go back home …because it was a lonely place without company*,* …which triggered my depression. Here*,* everyone cares about each other*,* and we do things together. We fold clothes together every day*,* chat like sisters*,* and feel like family. Overall*,* I’m living a comfortable life here.”*

**Case 4:**

*“She kept screaming*,* and everyone got to know her. …As an old man*,* I understand that aging can be uncomfortable. …If someone talks to her*,* it can help prevent her from yelling.”*

**Case 7:**

*“If no one feeds her*,* I will provide for her; I treat them as if they are our own parents. …Some of them eat messily*,* but I feed them*,* and they eat the meal cleanly. …she does not like it when others serve her. …I feed her*,* and she is pleased …after I feed her*,* I feel relieved*,* and then I return for my meal. I do it every day.”*

## Discussion

This study explored the experiences of residents without dementia who are living with those with dementia in long-term care facilities. Three themes were identified. The first theme, “the impact of co-living,” revealed that residents without dementia may feel helpless by the disruptions, such as noise and restlessness, caused by the BPSD of residents with dementia. If these problems persist and are not resolved, residents without dementia may feel increasingly helpless and unhappy.

This study found that residents without dementia did not react negatively or abuse residents with dementia because of their BPSD. This may be because the participants may not have been willing to actively inform the researchers or intentionally concealed these non-compliant behaviors.

The co-living experiences of participants in this study are aligned with Oh’s [19] investigation of the experiences of residents without dementia living with residents who had a stroke and dementia in nursing homes. Over time, respondents living with those with dementia gradually become irritable and frustrated, transitioning from sympathy to impatience. Furthermore, negative emotions such as helplessness and anger have similar impacts because recognizing the emotions cannot change the situation.

According to the second theme, “facing difficulties and coping,” residents without dementia may adopt various coping mechanisms when confronted with the BPSD of residents with dementia. Some may resort to passive avoidance to prevent being disturbed again or ignore the interference of residents with dementia to avoid distress. Others may take a positive approach and directly protest, refusing to endure it. Isaksson et al. [20] highlighted that caregivers of residents in nursing homes encounter similar situations and often feel a loss of control and helplessness when confronted with violent behavior from residents. These emotions may lead caregivers to maintain a distance from residents who exhibit violent behavior.

For the senior citizens, the relocation to a long-term care facility is to minimize the burden on their families. They value tailored care and fear losing autonomy and the ability to care for themselves which is the main reason for resisting adaptation [21]. The long-term care facility will be their home for the rest of their lives, and they look forward to recuperating there and engaging with other residents. Their daily routines and quality of life may be affected by the behaviors of residents with dementia. Therefore, when they cannot tolerate the behavior of residents, they take positive actions to speak up for themselves and fight for their rights.

This study found that some residents without dementia could rationalize and accept the mental and behavioral conditions of residents with dementia by changing their thoughts. They empathized with others to accept the difficulties of co-living with residents with dementia. This is similar to the findings of Isaksson et al. [22], who pointed out that caregivers in nursing homes, such as nurses and aides, attempt to find reasons for the violent behavior of residents in the institution. They attribute this behavior to old age, brain injury, and cognitive impairment. Furthermore, Ostaszkiewicz [23] found that nursing staff in long-term care institutions attempt to understand the BPSD of residents with dementia and communicate with them. This study found that coping behaviors were similar and that residents without dementia regard living with residents with dementia as both “fate” and “cause and effect.” This may be related to karma and destiny in Buddhist and Taoist cultures in China. Suffering is believed to eliminate karma. Helping people with dementia can also have merit. They feel that they should not blame people for having dementia, and rather face the situation and accept them calmly. It is the cause and effect of their past lives and must be tolerated in this life.

The third theme, “companionship and reciprocity,” revealed that residents without dementia experienced the meaning of existence through helping others and being needed by assisting residents with dementia in daily care such as feeding and accompanying them to use the toilet. Huang [24] highlighted that residents in nursing homes defined their life satisfaction by observing and understanding the lives of other residents. By participating in activities such as helping residents with laundry and cleaning, they recognized their positive contributions to the environment, perceived their value in the group, and gave them tasks and missions. In other words, altruistic behavior can increase positive meaning in life and happiness.

Cipriani [25] also found that residents who engage in altruistic behaviors such as praying for others, assisting with tasks such as pushing wheelchairs, or sharing gardening results, may feel needed and valuable. This, in turn, contributes to a sense of meaningfulness and improves their quality of life. The results of this study showed that when residents without dementia and those with dementia live together, in addition to the negative emotions caused by the BPSD of residents with dementia, a positive reciprocal relationship also exists. Kumar et al. [26] also found that the value of altruistic behavior among older adults increases their happiness, which can improve their health.

This study showed that residents without dementia observed that those with dementia who were offered more interaction and stimulation could calm their emotions and reduce their shouting behavior. This is different from the findings by Hsu et al. [27] who found that in long-term care institutions, the frequency of memory and behavioral problems of residents with dementia living in a mixed-shelter (non-solitary housing) is higher than that of residents living in separate rooms and that it is positively correlated with the number of people living in a room.

Hsu et al. [27] believed that more complex cohabitation patterns would make residents with dementia irritable and more likely to cause BPSD. Mixed care for patients without and with dementia in general long-term care facilities is the most common care model in Taiwan. Cheng’s research showed that cognitively intact residents co-living with residents with dementia attempt to seek harmony [13]. Our study found that the co-living of residents without dementia and residents with dementia has the disadvantage of life interference. However, reciprocal advantages also exist in the interaction between the two and cannot be ignored.

Nygaard et al. [28] highlighted that when a person with dementia moves into a nursing home and must regard the nursing home as “home,” their feelings focus on “me and my relationship with other residents.” They hope to establish meaningful relationships within the group. The residents who live with them can be both a resource and a burden, making them feel either safe or insecure. Arai et al. [29] showed that residents with dementia in long-term care institutions who participate in less social activities and communications with other residents have severe BPSD. This demonstrates that social interaction plays a vital role in the health of residents with dementia.

However, as shown by the current study, attention must be paid to the fact that when people with dementia and those without dementia live together, two situations may occur: coming together and pulling away. When residents without dementia face interference from the BPSD of residents with dementia without appropriate intervention, it may increase the social distance, complaints, boredom, or intolerance symptoms, and lead to physical, social, and emotional separation between residents [30]. In other words, residents may be caught between “facing difficulties and coping” and “companionship and reciprocity.” As such, those who cannot cope effectively cannot enter the “companionship and reciprocity” stage. The only way to reduce conflicts is by separating or isolating residents with dementia. We should consider how to reduce conflict through environmental planning and professional intervention, supporting residents without dementia in adapting to and getting along with residents with dementia by sympathizing, caring, and sharing, and encouraging them to gradually form beneficial and friendly social interactions.

The Service-Integrated Housing (SIH) care model has gained popularity in recent years because it caters to the diverse needs of residents in institutions. These needs include different age groups, chronic illnesses, and cognitive impairments. The SIH model implements symbiotic care that involves residents and uses their skills and abilities to assist co-residents in a facility. This form of symbiosis helps meet residents’ physical, emotional, and social health needs. Meyer et al. [31] stated that symbiotic care involves four key attributes: cohabitation, non-peer interaction, mutualism, and agency sponsorship. These attributes are consistent with the results of the present study.

This study also found that in long-term care institutions, residents without dementia and residents with dementia live closely together. The residents have unique and complementary needs and abilities. Through co-living activities, residents experience mutual benefits. However, long-term symbiotic care to meet residents’ healthcare needs and improve their quality of life continues to attract the attention of governments and institutions. Symbiotic care also requires further clinical application and evaluation of its effectiveness in the future.

### Clinical implications

In long-term care facilities that house residents with and without dementia, staff must be attentive to how residents with dementia and their BPSD can affect the lives of those without dementia. To ensure that all residents live comfortably, it is crucial to implement policies to include temporary living arrangements and available rooms. Encouraging residents with better abilities in daily activities to assist those with impaired functions can promote mutual assistance and interactions among residents.

Support by residents without dementia is highly recommended to help them understand the needs and challenges faced by residents with dementia. This can lead to a mutually beneficial experience for both groups and inspire residents without dementia to engage in acts of kindness, thereby adding value to their lives. It can also encourage more social activities and facilitate the rehabilitation of residents with dementia, which can enhance cognitive function and slow BPSD.

The Taiwanese government’s efforts in the field of long-term care began with the promulgation of the “Long-term Care Services Act” in 2015, which defines that the subject of care is no longer limited to the senior citizens, but is considered by a degree of physical and mental disability. To further solve the problem of a super-aged society, the “Long-term Care Services 10-year plan (2017–2026)” is attempting to develop the overall care service system of the community [14] and incorporate the Japanese concept of multi-care in co-living communities. It is hoped that this will ignite the community’s ability to support and care for each other [32]. This study describes the symbiotic experiences of residents without dementia and with dementia as companions in a reciprocal relationship similar to the principles of co-living communities. In addition to supporting government policies, this study also serves as a tool for promoting sustainable care and harmonious living.

### Strengths and limitations

Interviews were conducted with 30 residents without dementia from three residential long-term care institutions located in central Taiwan. Due to the varying courses of illness, cognitive abilities, and BPSD, the challenges and impacts of co-living in the same housing environment are complex and varied. The interviewed participants shared their personal characteristics, living experiences, and unique perspectives on co-living with residents with dementia. Therefore, the findings cannot be generalized to all the co-living situations of residents without dementia and residents with dementia in general institutions.

Additionally, although we excluded residents with mental illness to focus on the experiences of patients who were cognitively intact, further study should include the experiences of such residents and the effects that such residents have on others.

Another limitation is that the participants may not have been willing to share with the researchers any of their extreme reactions (even abuse) to patients with dementia. This may have influenced the findings.

Further, the research institutes were all private and the management may differ from public institutions which may affect the universality of the results.

## Conclusion

Living environments can significantly impact health. When individuals without dementia live with patients with dementia, it can affect their lives. The BPSD of residents with dementia can cause interference, distress, and negative emotions. However, residents without dementia may gain meaning and self-validation by interacting with residents with dementia. To ensure positive social interactions and friendships in long-term care institutions, staff should promptly and appropriately intervene. This can enhance the advantages and reduce the disadvantages of mixed living. Residents may even improve their life satisfaction and health in their later years through altruistic behavior.

## Data Availability

The datasets used in this study are available from the corresponding author upon request.

## Abbreviations

BPSD: Behavioral and psychological symptoms of dementia
DCM: Dementia care mapping
DSCUs: Dementia specialty care units
ADL: Activities of daily living
IADL: Instrumental activities of daily living
MMSE: Mini-mental state examination
SIH: Service-integrated housing
